# Therapeutic Advances in Multiple Sclerosis

**DOI:** 10.3389/fneur.2022.824926

**Published:** 2022-06-03

**Authors:** Jennifer H. Yang, Torge Rempe, Natalie Whitmire, Anastasie Dunn-Pirio, Jennifer S. Graves

**Affiliations:** ^1^Department of Neurosciences, University of California San Diego, San Diego, CA, United States; ^2^Department of Neurology, University of Florida, Gainesville, FL, United States

**Keywords:** multiple sclerosis, disease modifying therapy, demyelination, neurodegeneration, review

## Abstract

Multiple sclerosis (MS) is an autoimmune disease affecting the central nervous system that causes significant disability and healthcare burden. The treatment of MS has evolved over the past three decades with development of new, high efficacy disease modifying therapies targeting various mechanisms including immune modulation, immune cell suppression or depletion and enhanced immune cell sequestration. Emerging therapies include CNS-penetrant Bruton's tyrosine kinase inhibitors and autologous hematopoietic stem cell transplantation as well as therapies aimed at remyelination or neuroprotection. Therapy development for progressive MS has been more challenging with limited efficacy of current approved agents for inactive disease and older patients with MS. The aim of this review is to provide a broad overview of the current therapeutic landscape for MS.

## Introduction

Multiple sclerosis (MS) is the most common autoimmune disease of the central nervous system (CNS) affecting >900,000 people in the United States and >2 million people worldwide (1, 2). Epidemiologically, MS is a heterogenous disease influenced by genetic factors, such as the association with HLA-DRB1^*^15:01, and environmental factors, including vitamin D level, obesity, smoking and Epstein Barr Virus (EBV) infection (3, 4). The diagnosis is made when patients present with a typical clinical syndrome coupled by evidence of lesion dissemination in space and time. The revised 2017 McDonald's criteria allows for earlier diagnosis in the setting of a single clinical attack and corresponding MRI findings of symptomatic or asymptomatic, enhancing T1 or non-enhancing T2 lesions typical of MS, and/or presence of cerebrospinal fluid (CSF) specific oligoclonal bands (5). The historical clinical subtypes include clinically isolated syndrome (CIS), relapsing-remitting MS (RRMS), primary progressive MS (PPMS), and secondary progressive MS (SPMS) (5). A CIS is defined as a first demyelinating episode with features typical of an MS attack such as optic neuritis, brainstem or spinal cord lesion, but not yet fulfilling full criteria for MS. A more recent refinement of MS disease subtype classification proposed by Lublin et al. is similar with the additional caveat of modifying MS subtypes as “active” or “not active” based clinical relapse and/or MRI activity (6). There is growing evidence that phenotype in MS (relapsing vs progressive) is likely driven by “host factors” most notably patient age, with younger patients having greater frequency of relapses and older patients more likely to have progressive phenotypes (7).

### Pathogenesis

Alterations in the peripheral immune system, blood brain barrier permeability, and intrinsic CNS immune cells (such as microglia) contribute to MS pathogenesis. Current therapeutic strategies target these three elements of MS pathogeny. Acute and chronic inflammation as well as neurodegeneration occur throughout the disease course, with prominence of acute inflammation in the relapsing phase of disease. The inflammatory process in MS has been studied in experimental autoimmune encephalomyelitis (EAE) animal models and pathological observations from patients with MS demonstrating the roles of both innate and adaptive immune responses (4, 8, 9). Innate immune cells with prominent roles in MS include myeloid-derived macrophages and microglia. Adaptive immune cells involved in MS include autoreactive CD4+ T cells, in particular Th1 cells, against myelin proteins and CD8+ cytotoxic T cells (10–12). Recent studies of specific T cell subtypes from MS patients demonstrated varying myelin targets that may correlate with different patterns of inflammation (13). Although B cells have not been shown to be critical for EAE in animal models, they play a key role in the pathogenesis of human MS by means of proinflammatory cytokine and chemokine production, antibody formation, and antigen presentation to T cells (14). The presence of oligoclonal bands (CSF-restricted IgG immunoglobulins) and antibody-complement depositions in MS lesions further implicate mature B cells in both relapsing and progressive forms of the disease (15). Though MS lesions are commonly recognized as focal areas of demyelination in white matter, the inflammatory injury also involves gray matter and the subpial/meningeal layers (9, 16). Progressive MS is suspected to result from cumulative injury due to chronic inflammation and neurodegeneration stemming from multiple pathogenic mechanisms including activated microglia, leptomeningeal inflammatory infiltrates causing subpial demyelination, and mitochondrial dysfunction and oxidative injury driven by macrophages and microglia (17, 18).

### Therapeutic Goals

There are no curative treatments for MS. Given the disease heterogeneity, there is no single therapeutic target for MS. The main goal of current disease modifying therapy (DMT) is to quiet the disease by reducing inflammation, myelin injury and relapses. A meta-analysis of various DMTs demonstrated that all studied DMTs reduced relapse rate within 2 years (19). Additionally, cohort studies have demonstrated that earlier treatment with a DMT reduced onset of disability with some suggestion that earlier high-efficacy therapy may be more successful at reducing disability than traditional therapies (20–24). Treatments for progressive MS remain elusive and challenging, further suggesting that the pathogenesis of MS evolves from the pro-inflammatory relapsing stage to the neurodegenerative stage of the disease less responsive to immune-based therapies.

**Figure 1:**
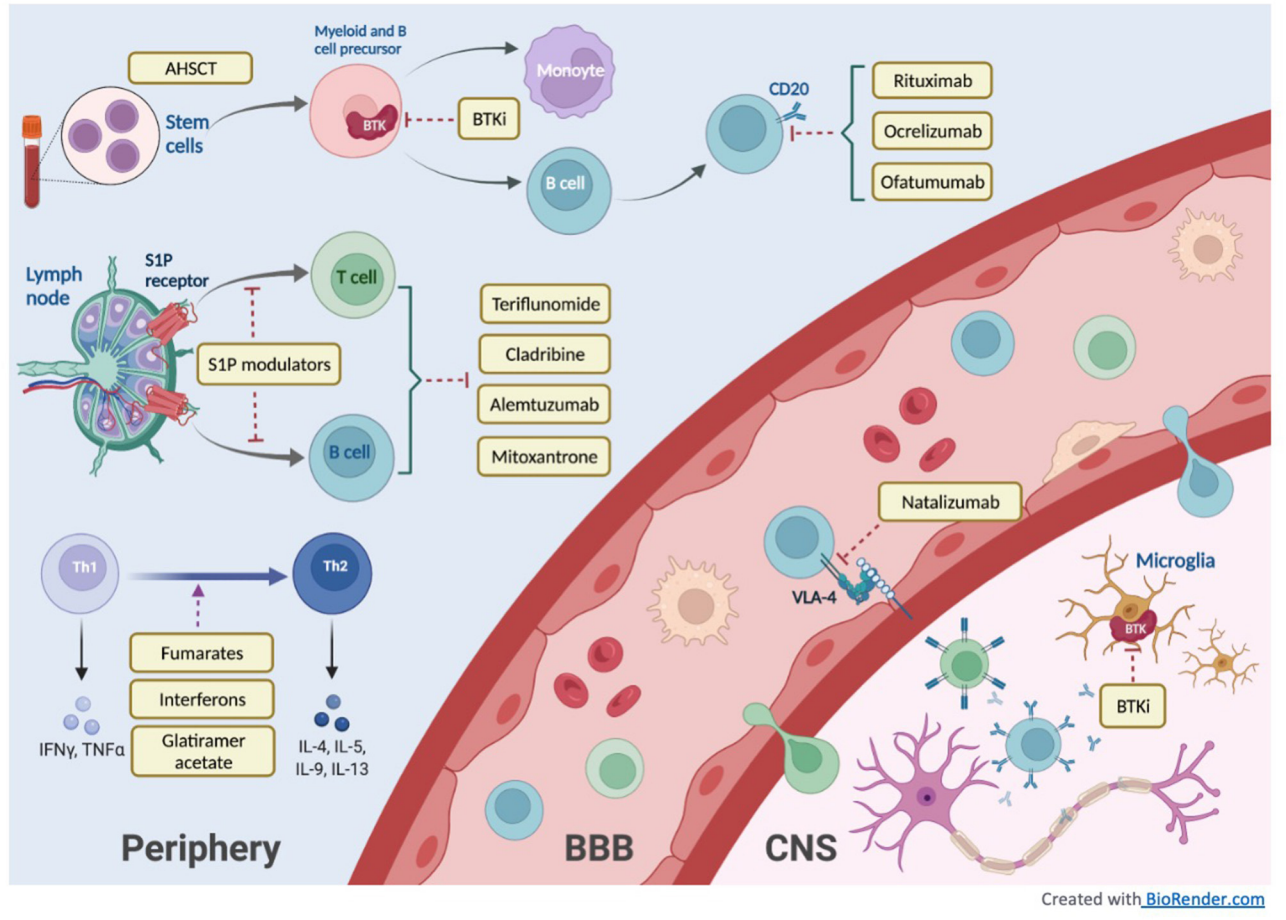
DMT mechanism of action.

In this review, we will discuss the therapeutic advances in MS treatment over the last 30 years with a focus on new high-efficacy DMTs as well as current developments in progressive MS treatment. Figure 1 illustrates the mechanisms of different DMT groups, and Table 1 summarizes their mechanisms of action along with dosing, pivotal clinical trials, adverse effects and laboratory monitoring recommendations. Table 2 summarizes the current recommendation for DMT use in pregnancy.

**Table 1:** Current therapeutic treatments for MS.

	**Medication/medication class**	**Mechanism of action**	**Route and dosing**	**Half life**	**Approved for:**	**Pivotal clinical trials**	**Adverse effects**	**Lab monitoring**
**Traditional injectables**								
*Interferons*	Interferon beta-1a (Rebif)	Immune modulation	SQ; 44 mcg 3x/week	69 ± 37 h	CIS; RRMS; Active SPMS	PRISMS	**Common**: Injection site reaction Flu-like symptoms Headache **Warnings:** Idiopathic thrombocytopenia Hyper/ hypothyroidism Rarely autoimmune hepatitis	**Baseline**: CBC, LFTs, TSH, TB, T cell subsets. **Routine**: CBC, LFTs q6 months
	Interferon beta-1a (Avonex)	Immune modulation	IM; 30 mcg 1x/week	10 h	CIS; RRMS; Active SPMS			
	Interferon beta-1b; (Betaseron, Extavia)	Immune modulation	SQ; 250 mcg QoD	8 min−4.3 h	CIS; RRMS; Active SPMS	IFNB; BENEFIT		
	Pegylated interferon beta-1a (Plegridy)	Immune modulation	SQ; 125 mcg every 2 weeks	78 h	CIS; RRMS; Active SPMS	ADVANCE		
	Glatiramer acetate; (Copaxone, Glatopa)	Immune modulation	SQ; 20 mg daily or 40 mg TIW	Unknown	CIS; RRMS; Active SPMS	GALA; PRECISE	**Common:** Injection site reaction Chest tightness Anxiety Lipoatrophy Skin necrosis	None required
**FDA-approved oral medications**								
*Fumarates*	Dimethyl fumarate; (Tecfidera)	Immune modulation	PO; Titrate up to 240 mg BID	1 h	CIS; RRMS; Active SPMS	DEFINE; CONFIRM	**Common:** Flushing GI upset **Warnings:** Lymphopenia PML (related to lymphopenia)	**Baseline**: CBC, LFTs, total bilirubin, T cell subsets, TSH, TB, pregnancy screen **Routine**: CBC, LFTs q6-12 months, T cell subsets if needed
	Diroximel fumarate; (Vumerity)	Immune modulation	PO; Titrate up to 462 mg BID	1 h	CIS; RRMS; Active SPMS	EVOLVE-MS2		
	Monomethyl fumarate; (Bafiertam)	Immune modulation	PO; Titrate up to 190 mg BID	0.5 h	CIS; RRMS; Active SPMS			
	Teriflunomide; (Aubagio)	Inhibition of cell division	PO; 7 or 14 mg daily	19 days	CIS; RRMS; Active SPMS	TEMSO; TOWER	**Common:** Headache Hair thinning **Warning:** Hepatotoxicity SJS/TEN Fetal malformations	**Baseline**: TB, pregnancy screen, BP, CBC, LFTs **Routine**: LFTs, CBC, BP monitoring
*S1P receptor modulators*	Fingolimod; (Gilenya)	Lymphocyte sequestration and altered cell migration; Binds to S1P receptor subtypes 1,3,4,5	PO; 0.5 mg daily; 0.25 mg daily if <40 kg; First dose observation required	6–9 days	CIS; RRMS; Active SPMS; Pediatric MS	FREEDOMS; TRANSFORMS; PARADIGMS	**Common:** Headache **Warnings:** Rebound syndrome Tumefactive lesions Macular edema Bradycardia/AV block Liver toxicity Hypertension Malignancy risk Seizures Fetal ris	**Baseline**: VZV IgG, OCT, CBC, LFTs, EKG, FEV1 if hx of COPD/asthma **Routine**: CBC, LFTs q6 months, OCT after 3-4 months, skin exams yearly
	Siponimod; (Mayzent)	Binds S1P receptor subtypes 1,5	PO; Titrate to 2 mg daily	30 h	CIS; RRMS; Active SPMS	EXPAND	**Warnings**: CYP2C9*3/*3 genotype	
	Ozanimod; (Zeposia)	Binds S1P receptor subtypes 1, 5	PO; Titrate to 0.92 mg daily	21 h to 11 days	CIS; RRMS; Active SPMS	SUNBEAM	**Common:** Nasopharyngitis headache URI **Warnings:** Untreated sleep apnea Concomintant MAOi use	
	Ponesimod; (Ponvory)	Binds S1P receptor subtype 1	PO; Titrate to 20 mg daily	33 h	CIS; RRMS; Active SPMS	OPTIMUM	**Warnings:** Bradycardia Hepatobiliary disorders Pulmonary events Macular edema Seizures	
	Cladribine (Mavenclad)	Cytotoxic effects on T and B cells by impairing DNA synthesis	PO; 3.5 mg/kg divided into two yearly treatment courses, each with 2 cycles Max 20 mg daily	24 h	RRMS; Active SPMS	CLARITY	**Common:** Headache URI HSV (prophylaxis needed if lymphocyte <200) **Warnings:** Lymphopenia Malignancy risk Fetal risk	**Baseline**: CBC, HIV, HBV, HCV, TB, VZV IgG, LFT, cancer screening **Routine**: CBC 2 and 6 months after each course and before 2^nd^ treatment
**Oral medications under investigation**								
*BTK inhibitors*	Evobrutinib	Myeloid and B cell depletion	PO; 25–75 mg daily	2 h	RRMS	Phase 2 completed	**Common:** Headache **Warnings:** Liver toxici	**TBD**
	Tolebrutinib		PO; 5–60 mg daily	2 h	RRMS; SPMS; PPMS	Phase 2b completed	**Common**: Headache URI Nasopharyngitis **Warnings**: Liver toxicity	
	Orelabrutinib		PO	Unknown	RRMS	Phase 2 under way	Not reported	
**High efficacy infusion and injectable medications**								
	Natalizumab; (Tysabri)	Altered immune cell migration via blocking α-4 β-1 and β-7 integrins	IV; SQ (Europe only); 300 mg q4-6 weeks	11 ± 4 days	CIS; RRMS; Active SPMS	AFFIRM; SENTINEL	**Common:** Headache **Warnings:** PML Rebound syndrome	**Baseline**: JCV Ab, CBC, LFT **Routine**: JCV Ab, CBC, LFT q6 months
*B cell depleting therapy*	Ocrelizumab; (Ocrevus)	CD20+ B cell depletion	IV; Induction: 300 mg day 1 and day 14; Maintenance: 600 mg q6 months	26 days	CIS; RRMS; Active SPMS; PPMS	OPERA I and II; ORATORIO	**Common:** Infusion reaction URI **Warnings:** Malignancy Hypogammaglobulinemia Infection risk PML	**Baseline**: TB, HBV, HCV, CBC, LFTs, B cell subset, immunoglobulins **Routine**: CBC, LFTs, B cell subset, immunoglobulins
	Ofatumumab; (Kesimpta)		SQ; Induction: 20 mg weeks 0, 1, 2; Maintenance: 20 mg q4 weeks	16 days	CIS; RRMS; Active SPMS	MIRROR; ASCLEPIOS I and II	**Common:** Injection site reaction URI **Warnings:** Infection Hypogammaglobulinemia	
	Alemtuzumab; (Lemtrada)	CD52+ T and B cells, natural killer cells, monocytes, macrophages	IV; **Year 1:** 12 mg/day daily x 5 days (total 60 mg); **Year 2:** 12 mg/day daily x 3 days (total 36 mg)	14 days	RRMS; Active SPMS	CARE-MS I	**Common:** Infusion reaction Headache **Warnings:** Hypo/hyperthyroidism Risk for autoimmune disease Strokes	**Baseline**: CBC, urinalysis, creatinine, TSH, VZV IgG, TB, HIV, skin exam **Routine**: CBC, creatinine, urinalysis monthly, and TSH q3 months, annual skin exam
	Mitoxantrone; (Novantrone)	Inhibition of cell division	IV; 12mg/m^2^ every 3 months; maximum cumulative dose 140 mg/m^2^	α: 6-12 min; β: 1–3 h; γ: 23–215 h; Median 75 h	RRMS; SPMS; PRMS	MIMS	**Warnings:** Myocardial toxicity Bone marrow suppression Malignancy risk	**Baseline**: CBC, LFT, echocardiogram, pregnancy testing
**Other available therapies**								
Autologous hematopoietic stem cell transplant		Immune cell reconstitution	—	—	RRMS; SPMS; PPMS	ASTIMS; MIST	**Warnings:** Early toxicity; Infertility; Secondary autoimmune disease; Myelodysplastic syndrome	—

*q, every; CIS, clinically isolated syndrome; RRMS, Relapsing and remitting MS; SPMS, secondary progressive MS; PPMS, primary progressive MS; IM, intramuscular; SQ, subcutaneous; PO, oral; IV, intravenous; GI, gastrointestinal; SJS/TEN, Steven Johnson Syndrome/ toxic epidermal necrolysis; PML, progressive multifocal leukoencephalopathy; URL, upper respiratory infection; ITP, idiopathic thrombocytopenic purpura; CBC, complete blood count; BP, blood pressure; LFTs, liver function tests; TSH, thyroid stimulating hormone; TB, tuberculosis; OCT, optical coherence tomography; VZV, varicella zoster virus; HIV, human immunodeficiency virus; HBV, hepatitis B; HCV, hepatitis C; JCV, John Cunningham virus; MAOi, monoamine oxidase inhibitor; TBD, to be determined*.

**Table 2:** Recommendations on DMT use during pregnancy (25).

**Medication/medication class**	**Safety in pregnancy**
**Traditional injectables**	
Interferons	No increased risk with early exposure
	Discontinue during pregnancy unless evidence of active disease
Glatiramer acetate	No increased risk with early exposure
	Safe to continue during pregnancy
**Oral medications**	
Fumaric acid derivatives	Discontinue at time of attempting pregnancy or positive pregnancy test
	Do not use during pregnancy
Teriflunomide	Black box warning due to teratogenicity
	Serum level should be <0.02 mg/L before attempting conception for women and men
S1P receptor modulators	Risk for congenital malformations
	Fingolimod: Discontinue ≥2 months before attempting pregnancy
	Siponimod: Discontinue ≥10 days before attempting pregnancy
	Ozanimod: Discontinue ≥3 months before attempting pregnancy
	Ponesimod: Discontinue ≥7 days before attempting pregnancy
	Consider bridging therapy due to increased relapse risk from discontinuation
BTK inhibitors	Unclear risk
	Not recommended during pregnancy at this time
Cladribine	Black box warning due to genotoxicity
	Last dose should be ≥6 months before attempting pregnancy
Mitoxantrone	Black box warning due to teratogenicity
**Infusion medications**	
Natalizumab	Risk for neonatal hematological abnormalities with ongoing exposure during pregnancy
	For active disease, may continue every 8 weeks until 34 weeks
	Not recommended during third trimester
	Consider switching therapy prior to pregnancy
Ocrelizumab	FDA recommend ≥6 months after last dose
	For active disease, last infusion should be 1–3 months before attempting pregnancy (half-life ~26 days)
	Delay contraception and re-dose DMT if pregnancy not achieved after 6–9 months
	Not recommended during third trimester
Ofatumumab (injectable)	Limited data but assume risk is similar to other B cell therapies
	FDA recommend ≥6 months after last dose
	For active disease, last dose should be 1–3 months before attempting pregnancy (half-life ~16 days)
	Not recommended during third trimester
Alemtuzumab	Last dose should be ≥4 months before attempting pregnancy
	Continue thyroid monitoring during pregnancy

## Early “Traditional” Injectable DMTS

### Interferon Beta

In 1993, interferon therapy was the first FDA approved DMT for MS. Current interferon beta therapies include both subcutaneous and intramuscular formulations with different injection frequencies ranging from every other day (interferon beta-1b) to every 2 weeks (pegylated interferon beta-1a). Interferon beta is a cytokine with several functions, which include downregulation of antigen presentation, thereby suppressing T cell activity, induction of IL10, which skews the differentiation of CD4+ T cells toward a Th2 phenotype, blockade of T cell migration by decreasing adhesion molecules and matrix metalloproteinase 9 (26, 27). Clinical trials assessing interferon therapy have shown to delay the onset of clinically definite MS in patients with CIS and reduce the severity and frequency of attacks (26, 28). The PRISMS randomized, double-blind, placebo-controlled trial demonstrated reduced relapse rate with interferon beta-1a (1.82 in the 22 microg group, 1.73 in the 44 microg group) compared to 2.56 in the placebo group with a relative risk reduction of 27–33% (29). In the ADVANCE phase 3 randomized trial for pegylated interferon beta-1a, there was a reduced annualized relapse rate in the treatment groups (0.256 in every 2 week group, 0.288 in every 4 weeks) compared to 0.397 in the placebo group, which resulted in a hazard ratio of 0.62 (95% CI 0.40–0.97) for both the every 2 week and every 4 week groups (30).

The PRISMS-15 study evaluated long-term outcomes of 290 patients enrolled in the original PRISMS study 15 years after initial randomization, 145 of whom had data on conversion to SPMS. The conversion to SPMS were lower in patients who had higher cumulative doses of interferon than those with lower cumulative doses, and each cycle of 5 years on interferon treatment, SPMS risk was reduced by 28% (HR 0.72, 95% CI 0.60–0. 86) (31). The main side effects of interferon therapy are injection site reactions and post-injection flu-like symptoms. Long-term safety data from the PRISMS-15 study demonstrated that 9–12% patients reported serious adverse events though the details of those adverse events were not reported. The annualized relapse rate was 0.50 (95% CI 0.46–0.54) for the lower cumulative IFN dose group, and 0.37 (95% CI 0.33–0.40) for the higher cumulative IFN dose group (31).

### Glatiramer Acetate

Glatiramer acetate (GA) was FDA approved in 1997 for the treatment of RRMS. It is a mixture of four amino acid polymers that bind to myelin-specific autoantibodies to reduce autoreactivity and promote a predominant Th2 phenotype (32). Although used originally to induce EAE in animal models, it was found to prevent EAE after injection of purified myelin basic protein (33). A phase III dose-comparison study demonstrated that both 20 and 40 mg GA dosing reduced the annualized relapse rate and mean number of gadolinium-enhancing lesions (34). GA has also been studied in primary progressive MS. Although a double-blind, placebo-controlled trial did not demonstrate a significant treatment effect in terms of the primary outcome of accumulated disability, it did significantly reduce T1 enhancing lesions at 1 year and T2 lesion burden at years 2–3 (35). Overall, GA is generally well-tolerated and safe to use during pregnancy (25). The main side effects include local injection reactions of the skin and more rarely panic-attack like episodes (flushing, chest pain, palpitations, dyspnea) that are usually transient and self-limiting.

Long-term outcomes for up to seven years for relapsing MS patients enrolled in the Glatiramer Acetate Low-frequency Administration (GALA) study demonstrated a 0.26 adjusted annualized relapse rate in the early-start GA group (randomized to GA in the placebo-controlled trial) compared to 0.31 in the delayed start GA group (switched to GA in the open label phase), with a risk ratio of 0.83 (95% CI 0.70–0.99, *p* = 0.04) with no differences between the two groups in terms of EDSS (expanded disability status scale) progression (36). The long-term safety profile was similar to those reported in the clinical trials, with the most common being injection site reactions (40%) and immediate post-injection reactions (12%); 11% patients had serious adverse events with 4 deaths that were deemed not related to the treatment drug (36).

### Interferons vs. GA

A multi-center, randomized head-to-head comparison of subcutaneous interferon beta vs. GA (REGARD study) did not show significant differences between the two drugs in time to first relapse (37). A real-world study of a large United States healthcare claims database using propensity score matching showed mildly lower annualized relapse rate comparing pegylated interferon 1a and GA (least square means ratio 0.809, 95% CI 0.67–0.97, *p* = 0.027) but no difference in healthcare resource utilization (inpatient stays p=0.83, durable medical equipment *p* = 0.29) (38). Another comparison using MSBase registry data using propensity-score matching demonstrated slightly lower relapse incidence in patients treated with GA and subcutaneous interferon beta-1a compared to intramuscular IFN beta-1a and IFN beta-1b (*p* < 0.001) though no differences in 12-month disability progression (39). Pegylated IFN was not included in the study.

## FDA Approved Oral Medications

### Sphingosine-1-Phosphate (S1P) Receptor Modulators

The S1P receptor modulator, fingolimod, was FDA approved in 2010 for the treatment of relapsing forms of MS. Since then, additional medications in this drug class have been approved, including siponimod (selective modulator of S1P1 and S1P5 receptors, contraindicated in patients with CYP2C9^*^3/^*^3 phenotype), ozanimod (selective modulator of S1P1 and S1P5 receptors), and ponesimod (selective modulator of S1P1). S1P is a phospholipid with five subtypes present in lymphoid tissue as well as endothelial cells, smooth muscles, atrial myocytes, spleen, and eyes. In the lymph nodes, S1P binding to S1P receptors is important for lymphocyte trafficking (40). S1P receptor modulator medications alter immune migration by binding to S1P receptors on lymphocytes, causing downregulation of S1P receptor expression and inhibiting lymphocyte egression from the lymph nodes (40).

In a phase 3, placebo-controlled double-blind trial (FREEDOMS II), there was a 48% reduction in annualized relapse rate for patients treated with fingolimod compared to placebo (rate ratio of 0.52, 95% CI 0.40–0.66) without a significant difference in disability progression (41). More recent trials with newer formulations include the SUNBEAM trial, comparing the safety and efficacy of ozanimod and interferon beta-1a which demonstrated lower annualized relapse rate in both 0.5 and 1.0 mg dosing with a 31% reduction in relapse rate, with a risk ratio 0.52 (95% CI 0.41–0.66) for the 1.0 mg dose and risk ratio of 0.69 (95% CI 0.55–0.86) for the 0.5 mg dosing (42). The OPTIMUM trial was a phase 3 study comparing ponesimod and teriflunomide, which demonstrated a relative reduction in annualized relapse rate by 30.5% (rate ratio 0.69, 99% CI 0.536–0.902) and a lower mean cumulative gadolinium enhancing lesions (relative risk 0.42, 95% CI 0.31–0.56) in the ponesimod group (43). Siponimod has been approved for both the treatment of RRMS and active SPMS (44).

S1P receptor modulators are associated with rare but serious side effects of macular edema, bradycardia, and AV blockade. Macular edema occurred in 0.3% of patients in clinical trials, which likely is the result of off-target effects, and patients with a history of diabetes uveitis or other risk factors for macular edema should avoid this class of medications (45). Earlier studies of less selective S1P receptor blockade such as fingolimod reported rare bradycardia and AV block, and patients are advised to obtain baseline EKG screening and assurance of no concomitant medications that affect heart rate or risk AV blockade prior to starting the medication. Fingolimod requires a 6-h first dose observation due to the risk for bradycardia as this risk is highest at 4–5 h. Treatment related adverse events from the SUNBEAM trial for ozanimod included nasopharyngitis, headache and upper respiratory tract infections without any clinically significant bradycardia or atrioventricular block (42). Treatment-related adverse events from the ponesimod OPTIMUM study were similar between the two arms, though more patients in the ponesimod group experienced bradycardia, hepatobiliary disorders, pulmonary events, macular edema and seizures (43). Because all S1P modulator drugs target the S1P1 receptor, which is expressed on lymph nodes as well as atrial myocytes, they all carry a theoretical cardiac risk. While newer formulations, particularly those with an up-titration over the first week, do not require a first dose observation period, the authors recommend exercising caution with screening for appropriate candidates for the S1P receptor modulators and warning patients against starting concomitant medications that can lower heart rates or cause AV blockade.

If fingolimod is stopped abruptly without effective transition to a new medication, rebound relapses with high number of enhancing lesions or tumefactive lesions can occur 4–16 weeks after discontinuation (46, 47). Furthermore, there is a low risk for the development of progressive multifocal leukoencephalopathy (PML) (48). As of August 2021, a total of 51 cases of fingolimod-related PML have been reported that were not related to prior natalizumab treatment (49). Risk factors may include duration of therapy (>18 months) and older age (>50 years), though lymphopenia is not strongly associated with PML cases, unlike in other immunotherapies (50). Several strategies to mitigate rebound risk after fingolimod discontinuation have been proposed, including monthly intravenous steroid pulses to bridge to the next therapy and shortening the washout period (47). The authors generally transition patients from an S1P modulator to another high efficacy DMT by discontinuing medication for 4–6 weeks, rechecking lymphocyte count at the end of the washout period, and immediately administering the first dose of the new DMT. Monthly steroid pulses are given if there are unexpected delays in initiating the new DMT.

### Teriflunomide

Teriflunomide was FDA approved in 2012, and it is currently approved for use in the treatment of relapsing forms of MS and active SPMS. Teriflunomide is the active metabolite of leflunomide that reversibly inhibits pyrimidine synthesis thereby preventing proliferation of activated T and B cells (51). The TEMSO randomized, double-blind, placebo-controlled study demonstrated a relative risk reduction of 31% in the annualized relapse rate as well as decreased disability progression and disease activity on MRI (51). Long-term efficacy from the 9-year TEMSO study follow up of 742 patients demonstrated an annualized relapse rate of 0.22 for the 14 mg BID dosing and 0.24 for the 7 mg BID dosing (52).

Teriflunomide should not be used in pregnancy or male or female patients planning a pregnancy as it may affect fetal development and remains in circulation for up to 2 years even after drug discontinuation. Accelerated elimination can be achieved with cholestyramine or activated charcoal. Additionally, teriflunomide carries a black box warning for severe liver injury; therefore, liver function testing should be monitored every month for the first 6 months and then every 6 months thereafter. Blood pressure and tuberculosis status should be checked before and after drug initiation. Other more common side effects include headache and hair loss. Long-term safety data from the TEMSO extension study reported 55% serious adverse events in the 14 mg BID dosing group and 62% in the 7 mg BID group; 11% of patients discontinued teriflunomide due to side effects, the most common being elevation in liver enzymes (52).

### Fumarates

Among the fumaric acid derivatives, dimethyl fumarate was first approved for MS in 2013. Other oral formulations include diroximel fumarate and monomethyl fumarate. The mechanism of action is the activation of the nuclear factor-like (Nrf2) pathway and improvement of anti-inflammatory responses by shifting cytokine production from interferon gamma and TNFα to IL4 and IL5 (53). A phase 3 placebo-controlled trial demonstrated a 44% relative reduction in annualized relapse rate with twice daily dimethyl fumarate, but no difference in time to disability (54). Exposure to dimethyl fumarate for up to 13 years in 1,736 patients enrolled in the DEFINE, CONFIRM and ENDORSE extension trials was associated with an overall annualized relapse rate of 0.15 (95% CI 0.118–0.194) (55).

The main side effects reported in this class of medications include gastrointestinal (GI) problems and flushing though the newer oral formulations in this class have potentially better GI tolerance (56, 57). About 30% of patients have reduced absolute lymphocyte count within the first year of treatment, and 2.5% of patients develop grade 3 lymphopenia, which increases their risk for PML or other severe infections. The PML risk is related to grade 3–4 lymphopenia (absolute lymphocyte count <500 x 10^6^/L) mostly affecting CD4/CD8 cells (48). As of September 2021, there are 12 confirmed cases of PML in dimethyl fumarate treated patients with an incidence of 1.07 cases per 100,000 person-years from post-marking reports (58). Most PML cases are linked to moderate to severe lymphopenia, but cases of mild lymphopenia have been reported (59). Thus far, there are no confirmed cases of PML in patients treated with diroximel fumarate. Long-term safety data from clinical trials have thus far reported 32% patients with serious adverse events, mostly MS relapses, with 14% discontinuing treatment due to other adverse events mainly related to GI intolerance (55).

### Cladribine

Cladribine is an oral medication FDA approved in 2019 for the treatment of relapsing forms of MS except CIS. It is a pro-drug that is phosphorylated to the active metabolite cladribine 2-cholordeoxyadenosine triphosphate causing intracellular accumulation of the metabolite and disrupting cellular metabolism, DNA synthesis and repair (60, 61). Unlike other oral DMTs, cladribine is not taken daily. It is dosed by weight (3.5 mg/kg), which is divided into two yearly treatment courses with two cycles per treatment course that are spaced 1 month apart. Cladribine disproportionately affects lymphocytes due to their high concentration of deoxycytidine kinase, which phosphorylates cladribine to the active metabolite, resulting in the apoptosis of CD4+ and CD8+ T cells as well as CD19+ B cells, while sparing other immune cells. The CLARITY study was a randomized, double-blind, placebo-controlled trial that demonstrated a lower annualized relapse rate (0.14–0.15 compared to 0.33 placebo) with a relative reduction of 54–57% (*p* < 0.001) in the two dosing groups compared to placebo, and the relapse-free rate odds ratio was 2.53 (95% CI 1.87–3.43) for 3.5 mg/kg dosing and 2.43 (95% CI 1.81–3.27) for the 5.25 mg/kg dosing (61).

Caution should be made when selecting patients for cladribine, as it does carry an increased risk for malignancy, specifically benign uterine leiomyomas, melanoma, pancreatic and ovarian carcinomas (61). However, in a meta-analysis of 11 phase III trials comparing the cancer rate of cladribine reported in the CLARITY and ORACLE MS trials vs. other DMTs (dimethyl fumarate, fingolimod, teriflunomide, natalizumab, alemtuzumab, glatiramer acetate), there were no significant difference in cancer rates among the various DMTs studied (62). In terms of safety monitoring, about 86% of patients develop lymphopenia ~2–3 months after initiation; therefore, close monitoring of lymphocyte count is indicated shortly after treatment.

## Oral Medications Under Investigation

### Bruton's Tyrosine Kinase Inhibitors (BTKi)

Bruton's tyrosine kinase inhibitors (BTKi) are emerging oral treatments for MS. BTKs are Tec family tyrosine kinases that are expressed in B cells, monocytes, neutrophils, and mast cells and are important for B cell maturation, proliferation, antigen presentation and differentiation to plasma cells. In myeloid cells (monocytes, granulocytes), BTK is important for cytokine and inflammatory mediator production and phagocytosis (63). BTK is also expressed in microglia, which are implicated in neuroinflammation of progressive as well as relapsing phenotypes, making it an attractive target for both forms of MS (64). Evobrutinib is a highly selective, irreversible oral BTKi shown in phase 2 trials to reduce T1 gadolinium-enhancing lesions without a significant difference in the annualized relapse rate between placebo and low and high Evobrutinib doses (65). In a phase 2b randomized, double-blind, placebo-controlled crossover study, Tolebrutinib, a CNS-penetrant BTKi, was shown to reduce T1 gadolinium-enhancing lesions in a dose-dependent manner at 12 weeks (1.03 placebo, 0.77 for 15 mg, 0.76 for 30 mg, 0.13 for 60 mg) (64). Orelabrutinib is another CNS-penetrant BTKi undergoing a phase 2 clinical trial for the treatment of RRMS. Several ongoing phase 3 clinical trials are underway and actively recruiting to investigate the efficacy of BTK inhibitors (clinicaltrials.gov: NCT04410991, NCT04410978, NCT04458051).

## High Efficacy Infusion and Injectable DMTS

### Natalizumab

Natalizumab is a monthly infusion DMT that was FDA approved in 2004. A subcutaneous formulation is approved for use in Europe but has not yet gained FDA approval in the United States. Natalizumab is a monoclonal antibody against the alpha chain in α4β1 integrin, also known as very late-activation antigen-4 (VLA-4), and the therapeutic mechanism of action is through inhibiting leukocyte infiltration across the endothelium into the brain. Phase 3 clinical trials (AFFIRM and SENTINEL) showed that natalizumab reduced clinical relapses at 1 year by 68 with 83% reduction in new T2 lesions and had better efficacy in combination with interferon beta-1a compared to interferon alone (66, 67). The Tysabri Observational Programme (TOP) using real-world data on the long-term efficacy of natalizumab reported an annualized relapse rate of 0.15 (95% CI 0.14–0.15) at 10 year follow up with lower relapse rates in participants with lower baseline EDSS scores <3.0. It is worth noting that in TOP these participants had exposure to fewer prior DMTs and fewer prior relapses (less active disease) (68).

Natalizumab manufacturing was briefly halted due to its link to PML which is related to JC virus reactivation as a consequence of impaired leukocyte migration and decreased T-cell mediated responses in the brain (69). However, PML risk can now be well stratified with the anti-JCV antibody index, duration of therapy and prior exposure to immunosuppressive medications (69, 70). The overall incidence of PML with natalizumab use is 4.14/1,000 patients, which has remained stable since the mid-2016 as risk stratification strategies became more widely incorporated into clinical practice (71). As of August 2021, the overall global incidence of PML in natalizumab treated patients was 3.75/1,000 patients (95% CI 3.50–4.0 per 1,000) (72). Data from the Phase 3b NOVA study recently showed that patients who switched to every 6 week dosing after 1 year of every 4 week treatment had similar efficacy in terms for relapse and disease activity compared to patients who remained on every 4 week dosing (73). Additionally, the retrospective analysis from the Tysabri Outreach: Unified Commitment to Health (TOUCH) program that included 35,521 patients suggested that there is also a reduction in PML risk with extended 6 week interval dosing (74).

If a patient becomes higher risk for PML due to a conversion to JC virus seropositivity, several transition strategies may be employed to transition to a different DMT though more studies are needed to determine safety and efficacy of the washout period. The switch to fingolimod has been studied in a double-blind, placebo-controlled trial demonstrating lower risk for MRI active lesions after an 8–12 week washout period compared to 16 weeks, and potentially decreased risk with a 4-week washout period compared to 8 weeks (75, 76). Several studies of patients with RRMS have demonstrated safe and effective transitions from natalizumab to anti-CD20 therapy (ocrelizumab or rituximab) (77, 78). A retrospective observational study from an Italian MS cohort evaluated annualized relapse rate, MRI activity and EDSS after patients transitioned from natalizumab (standard and extended interval dosing) to either ocrelizumab, rituximab or cladribine. The washout period for ocrelizumab was 8 ± 4.2 weeks, rituximab 7 ± 3.9 weeks, and cladribine 6 ± 2.9 weeks. The estimated annualized relapse rate of 0.001, 0.308, 0.5 respectively for ocrelizumab, rituximab and cladribine (no confidence intervals were given for these point estimates), and no significant difference between ocrelizumab and rituximab (77). The authors generally transition patients to another high efficacy DMT after natalizumab with a 4-week washout period, similar to other practice recommendations (79).

### Alemtuzumab

Alemtuzumab was FDA approved in 2014 for MS treatment. Two treatment cycles are completed 12 months apart with the first cycle consisting of 5 infusions and the second cycle consisting of 3 infusions. It functions by binding to CD52, a cell surface antigen on T cells, B cells, natural killer (NK) cells, monocytes, and macrophages. Following antibody binding to CD52, cellular lysis is induced by antibody-dependent cytolysis and complement-mediated mechanisms (80). In a phase 3 randomized controlled trial (CARE-MS I) comparing alemtuzumab to interferon beta-1a, patients treated with alemtuzumab had a 54.9% improvement in relapse rape with a 78% relapse free rate at 2 years (80). Additionally, 11% patients in the interferon arm had sustained disability accumulation compared to 8% in the alemtuzumab arm with a hazard ratio of 0.70 (95% CI 0.40–1.23, *p* = 0.22) (80). Long term efficacy over 9 years from the CARE-MS I and II trials showed that 62% (95% CI 54–69) were free of 6-month disability worsening and 50% (95% CI 41–59) had confirmed 6-month disability improvement (81).

Alemtuzumab requires long-term monitoring due to its risk for secondary autoimmune diseases (Graves' disease, immune thrombocytopenia, anti-glomerular basement membrane disease), malignancy (thyroid cancer, melanoma, lymphoproliferative disorders), bone marrow suppression and strokes (80). The risk for secondary autoimmunity could be related to higher serum IL21 levels with higher T-cell apoptosis and cell cycling (82). Other explanations for secondary autoimmunity are the early re-constitution of immature B cell types that are unchecked by T cells, and the imbalance between pro-inflammatory and regulatory lymphocyte subsets (83, 84).

### Rituximab

B-cell depleting therapy has emerged as a popular high efficacy class of MS medications. Rituximab is a monoclonal antibody targeting CD20, a cell surface marker of pre-B and B cells. Although not FDA-approved, rituximab has been commonly used off-label for almost 20 years. In a phase 2, double-blind trial in RRMS, patients who received rituximab had significantly reduced gadolinium-enhancing lesions and reduced relapses at 24 weeks (34.3% placebo and 14.5% rituximab) with a relative risk of 2.3 (90% CI 1.3–4.3) and 48 weeks (40% placebo and 20.3% rituximab, relative risk 1.9, 90% CI 1.1–3.2) (85). In a retrospective cohort study on prospectively collected data of 494 patients in Sweden, patients who received rituximab had significantly lowered relapse rate compared to injectable DMTs and decreased gadolinium-enhancing lesions compared to injectable DMTs and dimethyl fumarate (86).

### Ocrelizumab

Ocrelizumab is an intravenous humanized monoclonal antibody therapy targeting CD20 that induces an antibody-dependent cytolysis and complement-mediated lysis of B cells (87). It was FDA approved in 2017 for the treatment of RRMS (87). The OPERA I and OPERA II clinical trials were two parallel phase 3 multicenter, randomized, double-blind studies comparing the efficacy of ocrelizumab to interferon beta-1a at 96 weeks, showing a lower annualized relapse rate for ocrelizumab in OPERA I (rate ratio 0.54, 95% CI 0.40–0.72) and OPERA II (rate ratio 0.53, 95% CI 0.40–0.71). Secondary endpoints for disability progression at 24 weeks demonstrated a pooled hazard ratio of 0.60 (95% CI 0.43–0.84) favoring ocrelizumab, a reduction in T1 gadolinium-enhancing lesions for both OPERA I (rate ratio 0.06, 95% CI 0.03–0.10) and OPERA II (rate ratio 0.05, 95% CI 0.03–0.09), and reduction in new T2 hyperintense lesions for both OPERA I (rate ratio 0.23, 95% CI 0.17–0.30) and OPERA II (rate ratio 0.27, 95% CI 0.13–0.23) (87). In the 5-year open label extension phase of pooled OPERA I and II for RRMS, patients who remained on ocrelizumab maintained a low annualized relapse rate (0.14, 0.13, 0.10, 0.08, 0.07 for years 1–5 respectively). Patients who switched from interferon beta-1a to ocrelizumab at the start of the open-label extension demonstrated a relative rate reduction of 52% (p < 0.001) and continued to maintain low relapse rates at years 3–5 with ocrelizumab (88). The Ocrelizumab Biomarker Outcome Evaluation study in patients with RRMS suggested that ocrelizumab reduced serum neurofilament light chain (NfL), CSF NfL and CSF B cells, and in patients with PPMS, CSF NfL (*p* = 0.012) and CXCL13 (*p* = 0.020) were reduced (89, 90).

Ocrelizumab is the only FDA-approved medication for PPMS with a positive phase 3 trial (ORATORIO) with modest but significant reduction of confirmed disability progression at 24 weeks, 29.6% ocrelizumab vs. 35.7% placebo (HR 0.75, 95% CI 0.58–0.98, *p* = 0.04) and a reduction in total T2 lesion volume (HR 0.90, 95% CI 0.88–0.92) (65). In the open-label extension phase of at least 6.5 study years, patients who received ocrelizumab earlier had lower disability progression than those who received placebo (EDSS difference of 13.1%, 95% CI 4.9–21.3) and time to requiring a wheelchair (7.4% difference, 95% CI 0.8–13.9) (91). Similarly, those who received ocrelizumab earlier had a lower total change in T2 lesion volume (0.45 vs. 13%, *p* < 0.0001) and T1 hypointense lesion volume (36 vs. 61% initially placebo, *p* < 0.0001).

### Ofatumumab

Ofatumumab is a monthly injectable subcutaneous therapy approved in 2020. It is a monoclonal antibody that also binds to CD20+ B cells resulting in B cell depletion. Distinguishing features from other CD20 therapies are its shorter half-life and its initial uptake into lymph nodes after subcutaneous absorption. Two major clinical trials have demonstrated its efficacy for the treatment of RRMS. The phase 2b MIRROR study demonstrated an overall 65% reduction in the mean rate of cumulative new gadolinium-enhancing MRI lesions compared to placebo after 12 weeks (rate ratio 0.36, *p* < 0.001) with *post hoc* analysis showing an accumulation rate of 0.07 to 0.25 (rate ratio 0.08–0.29 compared to placebo, *p* ≤ 0.02) (92). The ASCLEPIOS I and II studies were phase 3 double-blind, double-dummy, randomized controlled trials comparing ofatumumab to teriflunomide. The annualized relapse rates were 0.11 for ofatumumab arm and 0.22 for teriflunomide arm in trial 1 (rate difference −0.11, 95% CI −0.16, −0.06) and 0.10 for ofatumumab and 0.25 teriflunomide in trial 2 (rate difference −0.15, 95% CI −0.2, −0.09). A pooled hazard ratio of 0.66 favored ofatumumab for disability worsening and 1.35 hazard ratio (95% CI 0.95–1.92) favoring ofatumumab for disability improvement (93).

### Safety and Monitoring Considerations in B Cell Depleting Agents

Side effects that should be considered for patients undergoing B cell depleting therapies include infusion reactions for rituximab and ocrelizumab, and injection site reactions for ofatumumab. All patients should have hepatitis (specifically HBV and HCV), HIV and latent tuberculosis (TB) screening prior to initiating therapy due to the risk for fulminant hepatitis and TB reactivation. Infusion-related reactions may include rash, itchiness, fever/chills, throat irritation, dyspnea, nausea, or headache, which can be improved with pre-treatment with diphenhydramine and methylprednisolone. The ENSEMBLE PLUS randomized study demonstrated similar rates of infusion related reactions in patients receiving the conventional 5–6 h infusion vs. a shorter 2 h infusion (proportional difference 2.44%, 95% CI −3.83, 8.71%) (94). Patients should be counseled on the risk for mild to severe infections as well as prolonged recovery from infections due to immunosuppression. There is concern for the risk of a potentially more severe COVID-19 course in MS patients treated with anti-CD20 therapy (95).

The long-term safety report for ocrelizumab up to 7 years for both relapsing MS and PPMS for adverse events were similar to the data from the phase 3 clinical trials. The rate per 100 patient years for serious adverse events was 7.3 (95% CI 7.0 −7.7), infusion reactions 25.9 (95% 25.9–26.6) and infections 76.2 (95% CI 74.9–77.4) with no increased risk for malignancy (96). Hypogammaglobulinemia from decreased IgG levels can occur after B cell depleting therapy, up to 30% in ocrelizumab, which can increase the risk for infections (97). Therefore, baseline then subsequent immunoglobulin levels should be obtained during therapy. Low immunoglobulin levels can be treated with maintenance intravenous or subcutaneous immunoglobulins if a critical level is reached–this level may differ by country but often considered to be <400 mg/dl.

In terms of lab monitoring, CD19, another B cell marker, is frequently used as a surrogate marker for CD20 B cell levels as the CD20 B cell therapies interfere with its direct detection in the blood. However, CD19 is expressed on a composite of B cell subsets, from immature to mature B cells, whereas the functional depletion of CD19+, CD27+ memory B cells are more important in reducing relapse in MS (98). Thus, another strategy is to monitor circulating CD27+ memory B cells in addition to CD19 counts, which can be ordered in commercially available B cell subset panels. CD19+ B cells are typically depleted within 2 weeks after rituximab and ocrelizumab and 12 weeks for ofatumumab. B cell repopulation occur around 6 months after infusion therapy, although some patients may have longer repletion periods and may be able to receive less frequent dosing, thereby reducing infection risk and allowing time for more effective vaccination responses (99). It is also important to note that certain T cell subsets also express CD20 and are depleted with anti-CD20 therapy, although the clinical significance of this is unknown (100). Additionally, anti-CD20 therapy, specifically rituximab, was found to partially reduce CD4+ and CD8+ T cells, though this reduction is transient (98, 101).

### Mitoxantrone

Mitoxantrone is an infusion therapy administered every 3 months that was FDA approved in 2000 for the use in RRMS and SPMS though it is rarely used today due to side effects. It functions by intercalating into DNA causing crosslinking and strand breaks thereby reducing the proliferation of T cells, B cells and macrophages as well as downregulating the inflammatory cascade (102). A double-blind, placebo-controlled trial in Europe (MIMS) demonstrated reduced mean number of relapses (0.4 compared to 1.20 in placebo) and reduced median time to relapse and decreased disability progression (102, 103). However, it is associated with cardiotoxicity and malignancy (specifically leukemia) thus falling out of favor compared to other commercially available DMTs (102, 104).

## Autologous Hematopoietic Stem Cell Transplantation

An emerging new therapy is autologous hematopoietic stem cell transplantation (AHSCT), which has been studied for the treatment of MS as well as other inflammatory disorders such as systemic sclerosis, Crohn's disease and neuromyelitis optica. The rationale for AHSCT is to “reset” the immune system. First, autologous peripheral blood stem cells are mobilized using cyclophosphamide and filgastrim and collected for post-ablation transplantation. Then, existing autoreactive immune cells are eliminated with either fully or partially myeloablative conditioning (chemotherapy) regimen (105, 106). Conditioning regimens may include cyclophosphamide (Cy) + anti-thymocyte globulin (ATG), Cy + alemtuzumab, or ATG + BEAM (carmustine, etoposide, cytarabine, melphalan) (106, 107). After conditioning, peripheral blood stem cells are re-infused to shorten the aplastic phase and reconstitute a new immune system with more regulatory cell types (regulatory T cells and CD56 NK cells) and decreased pro-inflammatory T-cell profiles (105, 108). Early toxicity is largely due to adverse reactions to the cytotoxic agents and myelosuppression. Late toxicity is more rare, but notable adverse effects include infertility, HSV, CMV and EBV reactivation, secondary autoimmune disorders and myelodysplastic syndromes (106).

There are as of this writing four retrospective studies, five single-arm clinical trials, and two randomized control studies evaluating the efficacy of AHSCT with promising results. In a meta-analysis of published studies using AHSCT for MS treatment, the pooled estimated transplant-related mortality was 2.1%, two-year disease progression rate was 17.1%, five-year progression rate of 23.3%, and a pooled 83% of patients with no evidence of disease activity at 2 years (109). Patients who had the most benefit and least mortality rate were patients with RRMS (106, 109). The two randomized controlled trials were the ASTIMS and MIST trials. ASTIMS was a phase 2 trial comparing AHSCT to mitoxantrone in 21 patients. None of the AHSCT-treated participants had T1 gadolinium-enhancing lesions after 4 years with an annualized relapse rate of 0.19 compared to 0.6 (in the mitoxantrone group rate ratio 0.36, 95% CI 0.15–0.88) (110). The MIST trial was a phase 3 crossover study comparing lower intensity non-myeloablative immunoablation (Cy + ATG) followed by a nonmyeloablative AHSCT to DMT (excluding ocrelizumab and alemtuzumab). The primary outcome result was a 93% reduction in the hazard for disease progression at 4–5 years in the AHSCT group compared to the DMT group (hazard ratio 0.07, 95% CI 0.02–0.24). It should be noted that the patients in the clinical trial had a higher level of disease activity at baseline, and the majority of the DMT group were maintained on lower efficacy DMTs (interferon, glatiramer acetate) (111).

A pooled treatment-related mortality from a meta-analysis is estimated to be 2.1%, though the mortality rate was 3.6% in patients who underwent transplantation prior to 2005, and only 0.3% in patients who underwent transplantation after 2005. Additionally, higher mortality was reported in patients who had more severe baseline disability scores (106, 107). Currently, there are several ongoing phase 2 and phase 3 AHSCT multi-center, randomized controlled trials comparing AHSCT to high-efficacy therapies such as alemtuzumab, natalizumab, ocrelizumab and rituximab (clinicaltrials.gov: NCT04047628 BEAT-MS, NCT03477500 RAM-MS). Both trials are enrolling patients with RRMS (age 18–55 for BEAT-MS and 18–50 for RAM-MS with high disease activity.

Overall, AHSCT seem to be a promising emerging therapy for MS especially as a one-time treatment rather than long-term immunosuppression. The patients who seem to benefit most are those with lower disability at baseline, more active/relapsing disease, and younger age (109). Therefore, at this time, AHSCT should generally be reserved for patients <45 years old with less disability, high disease activity, few or no comorbidities who have failed 1–2 high efficacy DMTs. The cost utility of AHSCT compared to DMT use should also be considered. Currently, the one-year cost of a myeloablative AHSCT regimen is approximately $181,933, most of which is incurred at the time of transplantation, and an adjusted $4,700 per quality-adjusted life year. Comparatively, the cost of DMT is approximately $70,000 per year due to rising costs of newer, higher efficacy DMTs with an estimated $73,000 per quality-adjusted life year (107). However, the data on the long-term effects on disease burden and morbidity/mortality with early AHSCT therapy are still limited (106).

## Pediatric MS

Pediatric-onset MS differs from adult-onset MS in that pediatric patients have more than three times higher relapse rates (112). Overall, 99% of pediatric patients present with relapsing forms of MS (112, 113). Few DMTs have been evaluated with completed double-blind, randomized phase 3 trials, but observational data have generally supported similar efficacy and safety of most DMTs in children <18 years old (114). DMTs that are frequently used include traditional injectables (interferon beta, glatiramer acetate), oral medications (dimethyl fumarate, teriflunomide), and infusion medications (natalizumab, rituximab and ocrelizumab). Prior cohort studies comparing injectable DMTs to newer higher-efficacy DMTs suggest that early treatment with newer DMTs result in lower relapse rate and lower rates of new/enlarging T2 lesions and gadolinium-enhancing lesions. In a multicenter cohort study of 741 patients in the United States Network of Pediatric MS centers, patients who received newer DMTs had fewer relapses with an adjusted annualized relapse rate of 0.22 compared to those who received traditional injectable DMTs with an annualized relapse rate of 0.49 (rate ratio 0.45, 95% CI 0.29 – 0.70) (115). In comparing oral to infusion DMTs, 25% relapsed on oral medications while 15% relapsed with infusions (115). A safety and efficacy study on the use of natalizumab in pediatric onset MS demonstrated in a small cohort of 19 patients a decline in median EDSS, with no new gadolinium-enhancing lesions during the treatment phase (116). Another phase 2 multi-center, non-randomized trial of dimethyl fumarate in 22 pediatric patients demonstrate a significant reduction in T2 hyperintense lesions after 8 weeks of treatment (117).

The first FDA approved DMT for the treatment of pediatric MS was fingolimod, which was studied in the PARADIGMS phase 3 randomized trial of patients ages 10–17 with pediatric onset MS with an absolute difference in annualized relapse rate of 0.55 (82% change) compared to interferon beta-1a (118). Safety profiles were similar between the two groups except for increased seizure frequency in the fingolimod arm (6 patients, 5.6%) compared to interferon (1 patients, 0.9%) (118). The TERIKIDS phase 3 randomized trial comparing teriflunomide and placebo did not show a significant difference in time to first relapse (hazard ratio 0.66, 95% CI 0.39–1.11, *p* = 0.29) though teriflunomide decreased the number of T2 lesions (RR 0.45, 95% CI 0.29–0.71), and T1 gadolinium-enhancing lesions (RR 0.25, 95% CI 0.13–0.51) (119). Pending pediatric trials include a phase 3 safety and efficacy study with dimethyl fumarate (clinicaltrials.gov: NCT02283853), and open-label safety and efficacy study with alemtuzumab (clinicaltrials.gov: NCT02283853). Currently, there is a phase 2 study and new phase 3 study on the use of ocrelizumab in pediatric patients, with the phase 3 trial comparing efficacy between ocrelizumab and fingolimod (clinicaltrials.gov: NCT04075266) and a phase 3 trial for siponimod and ofatumumab vs. fingolimod (clinicaltrials.gov: NCT04926818).

## Treatments for Progressive MS

### Pathogenesis

Primary progressive MS is defined as progression and worsening of disability from onset of disease without a clear history of relapses, while secondary progressive MS is defined as progressive accruement of disability with a prior history of relapsing disease (6). Progressive MS is further classified into active vs. non-active disease based on evidence of active disease (gadolinium-enhancing lesions) on MRI (120). The average onset of progressive MS is about 20 years after onset of relapsing-remitting disease while about 10–15% of MS patients present with primary progressive disease with rapid accruement of disability (121). The pathological features of progressive forms of MS include brain atrophy, cortical demyelination with subpial involvement, slowly expanding lesions, diffuse injury related to microglial activation in both gray and white matter, and meningeal lymphoid follicle-like aggregates composed of B cells within the subpial lesions (122, 123). In the neurodegenerative phase, acute inflammatory processes are less prominent, while chronic active and slowly expanding lesions predominate. Chronic active lesions demonstrate accumulation of extracellular iron in the oligodendrocytes in an age-dependent manner (122). Reactive oxygen species and nitric oxide from microglia and mitochondrial injury and dysfunction also contribute to progressive MS pathology. Secondary mitochondrial injuries are caused by chronic oxidative stress, and cortical neurons develop energy failure from decreased respiratory chain function and changes to the mitochondrial DNA. Progressive MS phenotypes have a clear association with chronological age given decreased relapse rate over time and greater accumulation of disability with increasing age (124). There is now growing evidence on the effects of biological aging on MS phenotype. As an example, shorter leukocyte telomere length associated with increased disability and progressive forms of MS in multiple cohorts (125–127).

### Clinical Trials Targeting Progressive Phenotypes

As discussed earlier, the only current FDA-approved DMT for PPMS is ocrelizumab. Several other phase 3 clinical trials involving DMTs in the treatment of progressive (primary progressive and secondary progressive) MS have resulted in positive results. These include interferon beta (reduced disability progression), mitoxantrone (reduced disease progression, relapse rate), ocrelizumab (reduced disability progression, brain volume loss), and siponimod (disability progression, relapse rate and functional scores) (44, 103, 123, 128, 129). However, efficacy was mostly seen in patients with active disease. A randomized double-blind, placebo-controlled trial with rituximab yielded similar results but no statistically difference in disease progression between the treatment arms. In a sub-analysis, disease progression was delayed in patients ages <51 years (hazard ratio 0.52, *p* = 0.010) and patients with active, gadolinium-enhancing lesions (hazard ratio 0.41, *p* = 0.007) (130). Data from the INFORMS phase 3 trial did not show a significant reduction in disability progression with fingolimod, and the ASCEND phase 3 trial with natalizumab did not reduce disability progression in patients with SPMS (131, 132). Currently, phase 3 randomized, double-blind trials are underway investigating the use of BTKi's for the treatment of PPMS (clinicaltrials.gov, NCT04458051) and SPMS (clinicaltrials.gov, NCT04411641).

In addition to DMTs, vitamin and co-factor supplementations have been studied for progressive MS treatment. Biotin, a coenzyme for five major carboxylases, was hypothesized to promote remyelination and neuroprotective mechanisms (123). However, while promising in phase 2, the phase 3 randomized, double blind, placebo-controlled international SPI2 study did not show significant benefit of biotin on walking speed or disability in PPMS or SPMS (133). Lipoic acid is an endogenous antioxidant that is involved in reducing oxidative species, iron chelation, and functions as a co-factor for pyruvate dehydrogenase and alpha-ketoglutarate dehydrogenase in mitochondria. A single-center double-blind, randomized phase 2 trial of 1,200 mg/day of alpha-lipoic acid daily demonstrated promising results with a 68% reduction in annualized percent change in brain volume with good safety and tolerability in patients with SPMS (134). A current phase 2 placebo-controlled trial is enrolling adult patients with progressive MS to study the effects of daily lipoic acid on the time 25 foot walk along with brain volume and other mobility measures (clinicaltrials.gov, NCT03161028). Recently, an exploratory study was published showing that N-acetylcysteine (NAC), a supplement that increases glutathione stores with neuroprotective potential, led to increased cerebral glucose metabolism and subjective improved cognition in MS patients (135). Low vitamin D levels have been associated with increased risk for developing MS, with some suggestion that level 25(OH)D levels predict slower progression of disease and lower T2 lesion volume, though the latter was mostly studied in patients receiving interferon therapy (136). A more recent cross-sectional study did not show a clear association with 25(OH)D levels with brain volume and progressive MS. However, vitamin D is postulated to exert an immunomodulatory effect and serve as a neuroprotective mechanism in progressive phenotypes (137).

Emerging cell-based immunotherapies are also being developed for progressive MS. A phase 1/2 study is underway for the safety and tolerability of ATA188, an allogenic Epstein Barr virus (EBV) T-cell therapy targeting EBV, which is well recognized risk factor for MS (clinicaltrials.gov, NCT03283826). This is based on the observation that defective CD8+ T-cell responses to EBV infection result in an accumulation of autoreactive B cells in the CNS that contributes to progressive MS pathology (138). An open-label phase 1 study with 5 SPMS and 5 PPMS patients demonstrated improvement in neurological function and quality of life and reduction in fatigue and intrathecal IgG production in six patients (139).

### Remyelination and Neuroprotective Strategies

Remyelination and neuroprotection in patients with MS are underway as additional therapeutic approaches. A phase 2b, multi-arm, double-blind, randomized, placebo-controlled trial in the United Kingdom studied the effect of three neuroprotective medications (amiloride 5 mg, fluoxetine 20 mg, riluzole 50 mg) on the percentage brain volume change at 96 weeks as well as T2 lesion burden, functional evaluations and time to first relapse in patients with SPMS, with no significant different in the primary or secondary outcomes (140). A previous randomized, double-blind study using clemastine fumarate to target oligodendrocyte precursor differentiation demonstrated improved latency delay using visual evoked potentials in patients with RRMS and chronic demyelinating optic neuropathy, suggesting that remyelination is possible following injury. The main side effect of clemastine was fatigue (141). A randomized, single-blind, parallel trial of 24 weeks of aerobic exercising with stationary cycling is underway to utilize somatosensory evoked potentials to measure functional remyelination in the spinal cord along with other functional outcomes such as timed 25-foot walk and 9 hole peg test (clinicaltrials.gov, NCT04539002). Metformin, a common medication used in diabetes mellitus, has been studied as a potential therapy to improve the regenerative potential of oligodendrocyte progenitor cells (142). A phase 1/2 randomized, double-blind trial will aim to investigate safety and tolerability of 3, 6 and 9 month dosing of metformin 500 mg/m2/day in children/young adults ages 10–25 years old in addition to optical coherence tomography and visual evoked potentials to investigate the effects of metformin on endogenous neuro progenitor cells (clinicaltrials.gov, NCT04121468). The SYNERGY trial using opicinumab, a monoclonal antibody targeting LINGO-1, a membrane protein that suppresses oligodendrocyte differentiation, was discontinued in a phase 2 trial due to not meeting primary and secondary endpoints.

## DMTs: When to Treat, How to Choose and When to Stop

Both the European Committee of Treatment and Research in Multiple Sclerosis (ECTRIMS) and the European Academy of Neurology (EAN) as well as the American Academy of Neurology (AAN) published guidelines in 2018 for the pharmacological treatment of people living with MS (143, 144). For CIS, the ECTRIMS/EAN committee recommended interferon or glatiramer acetate for patients with abnormal MRI suggestive of MS though not fulfilling full criteria for MS, though the AAN supports annual imaging for the first 5 years prior to initiating DMTs to screen to new disease activity. For confirmed relapsing MS, the recommendations from ECTRIMS/EAN and AAN align with the practices of most MS centers. Patients should be presented with all reasonable DMT options for their individual case taking into consideration their medical co-morbidities, disease severity, specific medication adverse effects, and medication adherence/ accessibility (143). In terms of relative efficacy among the various DMT options, although there are no head-to-head trials comparing all available DMTs, several studies have attempted to assess real-world efficacy among the various medications to decrease relapse rate and delay conversion to SPMS in relapsing MS patients.

### Comparing Oral DMTs

Real-world data comparing efficacies of oral DMTs may offer further insight into DMT of choice when discussing options with patients. A 2-year prospective study of 1,770 patients with RRMS from the French Multiple Sclerosis Registry demonstrated similar efficacy between teriflunomide (TRF) and dimethyl fumarate (DMF) using an inverse probability weighing on propensity scores, with 30.4% (95% CI 26.9–33.9) patients who experienced at least one relapse at 2 years in the TRF group vs. 29.5% (95% CI 26.6–32.2) in the DMF group and an odds ratio of 0.96 (95% CI 0.78–1.19) comparing DMF versus TRF (145). However, the adjusted proportion of new T2 lesions were lower in the DMF group compared to TRF (OR 0.60, 95%CI 0.43–0.82), and fewer patients withdrew from treatment due to lack of effectiveness in the DMF group compared to TRF (OR 0.54, 95% CI 0.41–0.74) (145). The cohort included RRMS patients who were either treatment-naïve or previously received injectable DMTs. Data from the Danish Multiple Sclerosis Registry compared TRF and DMF at 48 months and found lower annualized relapse rate for DMF (OR 0.58, 95% CI 0.46–0.73) and a lower incidence of discontinuation with DMF due to inefficacy (146). In the Italian MS cohort, findings at 38 months comparing DMF and TRF showed similar time to first event for TRF and DMF (HR 0.73, CI 0.52–1.03) but higher relapse-free survival in the DMF group after 38 weeks, and discontinuation rates between TRF and DMF at 24 months were comparable (147, 148).

### Injectable vs. Oral DMTs vs. High-Efficacy DMT

A multi-center retrospective study from the Italian MS Register compared the relapse rate, time to first relapse in 3,919 patients treated with first-line injectables DMTs (IFN or GA) to 683 patients treated with first-line oral DMTs (dimethyl fumarate or teriflunomide) (149). In this study, the oral DMT group demonstrated a lower time to first relapse (HR 0.57, 95% CI 0.47, 0.69) and annualized relapse rate (0.65 incidence ratio, 95% CI 0.52, 0.82) but no difference in the disability progression between the injectable and oral DMT groups (149). A comparative effectiveness study used an MS research registry and the electronic health record of 1,535 patients in the registry to determine 1-year, 2-year relapse rate and time to relapse for patients treated with dimethyl fumarate, fingolimod, natalizumab and rituximab after adjusting for confounders and propensity scores (150). The study compared natalizumab with rituximab, and dimethyl fumarate with fingolimod. Based on their statistical algorithms, there was an increased 1-year (0.08 rate difference), 2-year (0.132 rate difference) and shorter time to related (0.903 rate difference) in natalizumab-treated patients compared to rituximab. Interestingly, there were no significant difference in relapses between dimethyl fumarate and fingolimod in all three outcome measures (150).

### Conversion to SPMS

An international cohort study of 1,555 patients from the MSBase studied the risk of conversion to SPMS in patients treated with interferon beta, glatiramer acetate, fingolimod, natalizumab and alemtuzumab (21). The results demonstrated a delay in converting to SPMS for all drugs compared to untreated patients with a HR of 0.71 (95% CI 0.61–0.81) and 5-year absolute risk 12% for those treated with either IFN or GA, HR 0.37 (95% CI 0.22–0.62) and 5-year absolute risk of 7% for fingolimod, HR 0.61 (95% CI 0.43–0.86) and 5-year absolute risk of 19% for natalizumab, and HR 0.52 (95% CI 0.32–0.85) with 5-year absolute risk of 7% for alemtuzumab. Additionally, when patients were escalated from IFN or GA to fingolimod, natalizumab or alemtuzumab within 5 years, the HR was 0.76 (95% CI 0.66–0.88) with a 5-year absolute risk of 8% (21). Data for B cell therapies were not available at the time of the study.

Although newer DMTs demonstrate higher efficacy compared to older injectable medications, it is still uncertain whether earlier initiation of high-efficacy DMTs will alter the natural history of non-relapse related disease progression since immunotherapy has been less successful in treating progressive forms of MS. Several ongoing studies are investigating these questions including the prospective traditional vs. early aggressive therapy for MS trials (clinicaltrials.gov; TREAT-MS, DELIVER-MS) studies, which randomizes patients to traditional first-line therapy vs. high-efficacy DMT. The TREAT-MS trial compares traditional injectable and oral medications to higher efficacy DMTs including ocrelizumab, natalizumab, alemtuzumab, rituximab, cladribine, and ofatumumab to evaluate disability progression. DELIVER-MS compares high efficacy DMTs (alemtuzumab, ocrelizumab, natalizumab, rituximab, ofatumumab) to traditional injectables or oral medications to evaluate brain volume loss.

### Discontinuing DMTs

Due to a decline in overall inflammatory activity as patients enter the neurodegenerative stage of the disease, the effectiveness of DMTs directed at active disease activity diminish over time especially in patients with sustained absence of further disease activity. Considerations for stopping DMTs include increasing risks of complications from medication side effects with older age, increasing medical co-morbidities, and costs/expenses required for continuation of DMTs (151). Currently, the available data for DMT discontinuation in the later stages of the disease are based on retrospective analyses. A meta-analysis of 38 clinical trials assessed DMT efficacy on disability progression using a regression model, demonstrating that higher efficacy DMTs are most beneficial in younger patients in the earlier stages of disease but have limited benefit in the patients >53 years (152). A retrospective observational study from the Cleveland Clinic of 600 MS patients who were >60 years old evaluated clinical outcomes of discontinuing DMT after exposure to DMTs for at least 2 years (153). In this study, 29.7% of patients discontinued DMTs, of which 89% of patients remained off treatment, and only one clinical relapse occurred among those who discontinued DMTs. Performance scales, timed 25 foot walk, and nine hole peg tests did not differ among those who remained on and off DMT (153). Results from DISCO-MS, a randomized prospective study of patients >55 years evaluating relapse rate, MRI lesion burden, quality of life, and performance scales from stopping vs. continuing DMTs, will hopefully provide further insight into this topic (clinicaltrials.gov: NCT03073603).

## Conclusion

Over the last three decades, there has been a rapid expansion of treatment options for MS as well as increasing efficacy of newer agents against relapses. Despite advances in our understanding of the biology of MS pathogenesis, there remains a scarcity of effective treatment for progressive disease. While newer DMTs have higher efficacy in reducing relapse rate and MRI disease activity, they also may carry higher side effect profiles due to increased levels of immunosuppression. Part of the difficulty in the management of MS is the heterogenous nature of the disease, which is influenced by environmental and genetic factors as well as the naturally adaptive and evolving nature of the immune system that changes with time and age. There is promising increased activity in the field for the development of neuroprotective and remyelinating therapies including mechanisms to support mitochondrial function and cell-based therapies targeting culprits of chronic inflammation. Additional therapeutic approaches include harnessing immunoprotective mechanisms such as supporting regulatory T-cell function and reparative microglial function. Further studies are needed to identify early risk factors for an increased inflammatory state, early neurodegeneration, or a combination of both. Early therapeutic interventions for both the neuroinflammatory and neurodegenerative aspects of the disease, used in tandem, will likely be key to further therapeutic advances and the ultimate goal of true remission of the disease.

## Author Contributions

JHY contributed to the conception and drafting of the manuscript. TR, NW, AD-P, and JSG contributed to the editing and approval of the final manuscript. All authors contributed to the article and approved the submitted version.

## Conflict of Interest

JY has received speaker fees for NeurologyLive. TR received grant funding from the National Multiple Sclerosis Society. JG has received grant or contract funding from Biogen, Octave, Novartis, and EMD-Serono and has served on advisory boards for Bayer and Genentech and has received speaker fees from BMS and Alexion. The remaining authors declare that the research was conducted in the absence of any commercial or financial relationships that could be construed as a potential conflict of interest.

## Publisher's Note

All claims expressed in this article are solely those of the authors and do not necessarily represent those of their affiliated organizations, or those of the publisher, the editors and the reviewers. Any product that may be evaluated in this article, or claim that may be made by its manufacturer, is not guaranteed or endorsed by the publisher.
